# Two-Dimensional Titanium Dioxide–Surfactant Photoactive Supramolecular Networks: Synthesis, Properties, and Applications for the Conversion of Light Energy

**DOI:** 10.3390/ijms23074006

**Published:** 2022-04-04

**Authors:** Harold Lozano, Sindy Devis, Juan Aliaga, Matías Alegría, Hernán Guzmán, Roberto Villarroel, Eglantina Benavente, Guillermo González

**Affiliations:** 1 Departamento de Química, Facultad de Ciencias, Universidad Nacional de Colombia, Bogotá 111321, Colombia; hilozanoz@unal.edu.co; 2 Facultad de Ciencias de la Salud, Instituto de Investigación Interdisciplinar en Ciencias Biomédicas, Universidad SEK, Santiago 7520317, Chile; sindy.devis@zonavirtual.uisek.cl; 3 Departamento de Química, Facultad de Ciencias Naturales, Matemáticas y Medio Ambiente, Universidad Tecnológica Metropolitana Santiago, Santiago 7800003, Chile; jaliaga@utem.cl (J.A.); matias.alegriag@utem.cl (M.A.); 4 Departamento de Química, Facultad de Ciencias, Universidad de Chile, Santiago 7800003, Chile; hernan.guzman@ug.uchile.cl; 5 Instituto de Física, Pontificia, Universidad Católica de Chile, Santiago 7830614, Chile; roberto.villarroel@uc.cl; 6 Programa Institucional de Fomento a la Investigación, Desarrollo e Innovación (PIDi), Universidad Tecnológica Metropolitana, Santiago 7750000, Chile

**Keywords:** titanium oxide, layered supramolecular hybrids, TiO_2_ nanocomposites, inorganic-organic hybrid semiconductors, photocatalytic activity, fatty acids polymorphism

## Abstract

The desire to harness solar energy to address current global environmental problems led us to investigate two-dimensional (2D) core–shell hybrid photocatalysts in the form of a 2D-TiO_2_–surfactant, mainly composed of fatty acids. The bulk products, prepared by two slightly different methods, consist of stacked host–guest hybrid sheets held together by van der Waals forces between alkyl carboxylate moieties, favoring the synergistic conjugation of the photophysical properties of the core and the hydrophobicity of the self-assembled surfactant monolayer of the shell. X-ray diffraction and the vibrational characteristics of the products revealed the influence of synthesis strategies on two types of supramolecular aggregates that differ in the core chemical structure, guest conformers of alkyl surfactant tails and type, and the bilayer and monolayer of the structure of nanocomposites. The singular ability of the TiO_2_ core to anchor carboxylate leads to commensurate hybrids, in contrast to both layered clay and layered double-hydroxide-based ion exchangers which have been previously reported, making them potentially interesting for modeling the role of fatty acids and lipids in bio-systems. The optical properties and photocatalytic activity of the products, mainly in composites with smaller bandgap semiconductors, are qualitatively similar to those of nanostructured TiO_2_ but improve their photoresponse due to bandgap shifts and the extreme aspect-ratio characteristics of two-dimensional TiO_2_ confinement. These results could be seen as a proof-of-concept of the potential of these materials to create custom-designed 2D-TiO_2_–surfactant supramolecular photocatalysts.

## 1. Introduction

Heterogeneous photocatalysis based on metal oxides has received increasing attention in recent decades, mainly because it appears as a promising approach to face the accelerated anthropogenic deterioration of the biosphere caused by population growth and global development, converting solar energy into chemical energy—useful, for example, in the production of clean fuels or the destruction of organic pollutants [1]. Light-driven semiconductor excitation consists of promoting an electron from its valence band (VB) to the conduction band (CB), creating an electron–hole (exciton) charge pair, capable of becoming oxidation and reduction centers, spatially separated on the surface of the particle. The potential of the photogenerated redox centers is determined by the absolute energy of the VB and CB, while the minimum light energy necessary for the phenomenon to occur corresponds to the energy difference between the CB and the VB, the bandgap (Bg) characteristic of the material. The fraction of absorbed light usable to produce work (the quantum efficiency of the process) corresponds to the rate of photogenerated charges that survive their annihilation either by recombination or reaction with their environment before reaching the surface of the particle [2]. Broadband semiconductors, particularly TiO_2_ due to its electrical and photophysical properties, chemical and photochemical stability, abundance, safety, and low cost, have been intensively studied [2,3,4] for environmental applications in aqueous media. However, the lack of absorption of visible light and the high recombination rate of pure TiO_2_ and similar semiconductors affect their quantum efficiency under sunlight, limiting their applications, so enormous efforts are devoted to overcoming such obstacles. 

Beyond the traditional modification of the intrinsic properties of crystalline nanostructured TiO_2_ through doping with other elements or the formation of heterojunctions with semiconductors with a lower bandgap and/or compounds with other materials, including carbonaceous materials [3], using the phases’ amorphous and structural defects to improve the photocatalytic efficiency of TiO_2_ is being considered as more and more relevant [4,5,6]. This scenario of metastable phases and interfaces dominated by more subtle interactions requires less drastic working conditions than those necessary to optimize the crystallinity of conventional catalysts. Organic and supramolecular species are widely and successfully used to stabilize high surface energy in titania nanocrystals and are also used as a template or support matrix to obtain, by calcination, TiO_2_ hybrid materials, hierarchically structured titania photocatalysts with controlled morphology, size, and aspect ratios [7,8]. However, the photocatalytic activity of these precursors as prepared has seldom been investigated, even though the photosensitivity of poly(hydroxyethyl methacrylate)-based TiO_2_ gel hybrids resulting in stable photochromic materials was reported relatively early on [9].

However, in recent years, titania supramolecular hybrid photocatalysts have been receiving increasing attention. For example, the dispersion of TiO_2_ nanoparticles (NPs) on microfibrillated-cellulose-based substrates and the decoration of nanowhiskers (CN) on CN–TiO_2_ core–shell materials [9] has led to flexible UV-active photocatalysts [7,8,10]. Supramolecular sensitization of TiO_2_ by thiolated β-cyclodextrin in the TiO_2_-Au NC–β-CD system has also been reported [11]. The growth of TiO_2_ crystals on cellulose scaffolds alters the bandgap, allowing the photoreduction of Cr(VI) under visible light [12]. Other supramolecular aggregates that promote visible-light-driven TiO_2_ photocatalysts are, for example, the core–shell structures of 4-methoxycalyx [7] arene-immobilized TiO_2_ nanoclusters [13], bound TiO_2_ NPs to the hydroxyl-substituted calyxes [4] arene/oligothiophene/triphenylamine [14], and the polyanionic molybdate shells [Mo_9_O_28_]^2−^ intercalated with [1,3-bis(4-methylpyridine)propane]^2+^ cations [15].

The self-assembly of long carbon chain amphiphiles is a powerful tool as a template for the preparation of two-dimensional (2D) mesoporous nanostructures [16,17,18] that, for example, have been widely used to obtain siliceous [19] and non-siliceous nanomaterials [20,21], including TiO_2_ [22]. Two-dimensional materials, due to their large surface interfaces and extended pore structures, uniformly distributed with virtually no diffusion barriers, are particularly suitable for catalyst designs [23,24]. However, examples of hybrid TiO_2_ photocatalysts based on the self-assembly of simple surfactants such as fatty acids are even fewer than the supramolecular semiconductors just discussed. This is despite the fact that, as previously reported [25], the modification of the surface of the TiO_2_ NPs with long-chain carboxylic acids and other surfactants—in addition to producing colloidal dispersions of titania in organic solvents—induces changes in the semiconductor properties. These products are a type of supramolecular core–shell material that enhances the photocatalytic efficiency of titania to partially degrade gaseous NOx under visible light [26]. Furthermore, as recently reported [27], the electric field created by grafting the fatty acid carboxylate group onto the surface of the titania should explain the enhanced visible-light-driven photodegradation of amoxicillin obtained with the catalyst, Ti/TiO_2_/fatty acid/ZnO/graphene oxide.

In this work, inspired by the preliminary results just mentioned [26,27] and the current trend to develop inorganic–organic hybrid photocatalysts, as well as new complementary experiments, we analyze the synthesis, structure, and properties of supramolecular semiconductor materials formed by stacking 2D TiO_2_ networks of subnanometric thicknesses, each stabilized by confinement between self-assembled monolayers of linear saturated fatty acids, TiO_2_–fatty acids. We accomplished this while paying special attention to the effect of the synthesis strategies on the characteristics of the products. The synergistic conjugation of the extreme aspect ratio of the oxide sheets with the amphiphilic nature of the organic moiety leads to hydrophobic laminar particles with semiconductor properties suitable for the conversion of light energy.

## 2. Results

### 2.1. Synthesis Strategies

Given the high thermal and kinetic stability and crystallographic 3D habit of titanium oxide polymorphs, obtaining 2D titania nanostructures implies using bottom-up nanochemistry procedures that simultaneously allow both control growth and surface passivation of the particles. Heterosupramolecular scaffolds of TiO_2_ hybrid sheets were prepared using the common sol–gel reagent titanium tetraisopropoxide (TTIP) as a titanium precursor, along with amphiphilic molecules such as alcohols or carboxylic acids playing the role of the reagent and director of the structure. The products were attained following two distinct synthesis strategies that mainly differed by the temporality of both metathesis and hydrolysis reactions that can occur and, consequently, we here call (and label) them sequential (A) and concurrent (B) processes, respectively.

### 2.2. Sequential Processes

**Scheme 1:**
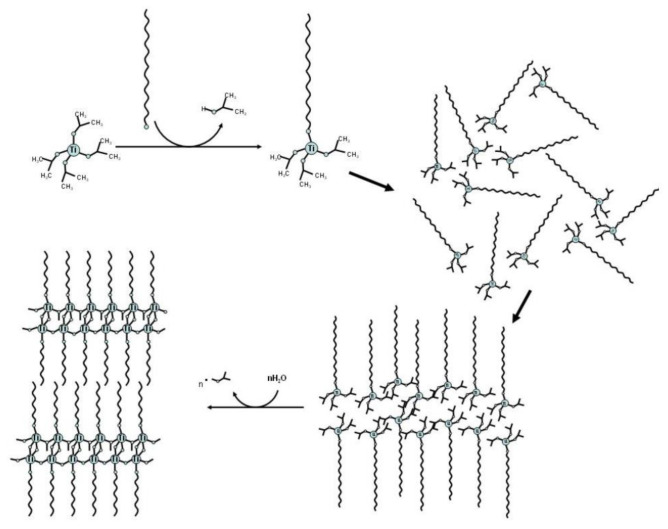
Sequential process for the preparation of laminar nanocomposites TiO_2_–organic-amphiphiles type A.

The sequential procedure, illustrated in Scheme 1, considers the following steps: (i) The preparation of hybrid amphiphiles via a double-replacement reaction of neat inorganic TTIP and organic (e.g., alcohols, carboxylic acids) precursors under an inert atmosphere (Ar); (ii) two-dimensional self-assembly and hydrolysis under air moisture of the hybrid organic–inorganic amphiphile. This strategy was employed to prepare a derivative of 3-butyn-1-ol (TiBTO) and n-hexadecanoic acid (palmitic acid, TiPA-A).

#### 2.2.1. Titanium Oxide–3-Butyn-1-Oxide (TiBTO) by the Sequential Process

**Figure 1:**
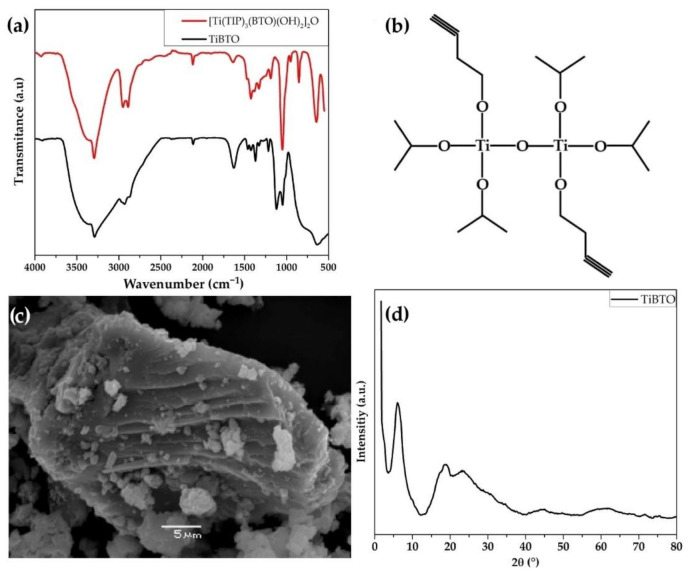
Infrared spectrum (FTIR) of TiBTO (**a**); scheme molecular dimer [Ti(TIP)_3_(BTO)(OH)_2_]_2_O (mp–TiBTO) (**b**); SEM micrograph (**c**); and diffractogram of TiBTO (**d**).

The product of the alcoholysis of pure TTIP with pure 3-butyn-1-ol at 70 °C under an argon atmosphere is a moisture-sensitive liquid (Scheme S1). The product was labeled as mp-TiBTO (the molecular precursor of TiBTO). As observed in the Fourier transform infrared spectrum (FTIR) shown in Figure 1a, the main 3-butynol absorption bands as the stretch modes ν(HC≡), ν(C≡C), and ν(CO) at 3293, 2177, and 1047 cm^−1^, respectively [28], and appear in the mp-TiBTO FTIR spectrum at the same positions. However, there are differences between both spectra that point to the conversion of 3-butynol to its alkoxide. Among them, the redshifts of the ν(CH) modes at 2951 and 2891 cm^−1^, the lack of broadband ν(OH) and the water traces (δ(H_2_O) at 1633 cm^−1^), and a new strong band at 1122 cm^−1^ assignable to the stretch mode ν(Ti-OC) [29] were observed in mp-TiBTO. Moreover, the spectrum also shows a band at 2968 cm^−1^ assignable to the ν(C-H) mode of the methoxide groups [29], as well as a wide absorption in the range of 3630–3350 cm^−1^ at low intensity. Since the presence of free TTIP is unlikely after the sample-purification treatment (vacuum distillation, 120 °C), we suggest the formation of a partially hydrolyzed titanium alkoxide oligomeric intermediate. Such an assignation is consistent with a rapid preliminary proton nuclear magnetic resonance (^1^H-NMR) measurement of an mp-TiBTO sample dissolved in CDCl_3_ (Figure S1). The spectrum indicates the coexistence of two types of alkoxide groups, isopropoxide (CH_3_ at 1.25 ppm and CH at 4.53 ppm) and 3-butynoxide (at 1.96, 2.58, and 4.36 ppm corresponding to the hydrogens HC≡, CH_2_, and OCCH_2_, respectively). Changes with respect to the spectrum of free 3-butynol in ^1^HNMR, such as the disappearance of the OH proton and resonance shifts, the high field shift of δ(HC≡), and the downfield shift of methylene protons (∆δ OCH_2_ > CH_2_), indicate the formation of 3-butynoxide. In turn, the broad resonance observed at 4.71 ppm would come from the presence of Ti-OH protons. This, together with the ratio of the area under the peaks for CH_3_ and HC≡ protons, approximately 12:1, suggests the formation of the dimer [Ti(TIP)_3_(BTO)(OH)_2_]_2_O (Figure 1b). Similar to what has been reported for the reaction of titanium tetrabutoxide with acetic acid [30], the reaction of TTIP with 3-butinol preferentially leads to monosubstituted titanates. This is relevant to the strategy used to prepare the supramolecular products described here. The advanced hydrolysis carried out by prolonged exposure to air of the molecular species generated from the partial alcoholysis reaction of TTIP with 3-butynol leads to the formation of an insoluble solid whose composition corresponds to the formula TiO_2_(C_4_H_5_)·0.25(TiO_2_ H_2_O) (Table S1). The equimolecular ratio TiO_2_:BTO is completely reproducible, but the amount of water and excess TiO_2_ that can vary slightly between different experiments are not. Since the product is a commensurate composite with defined stoichiometry, we will refer to this material and the similar ones described later as nanocomposites. FTIR spectra of TiBTO confirm the presence of an organic butyn-O- moiety in the composite linked by Ti-O-C bridges. Specifically, the vanishing of bands at 1047 cm^−1^ assignable to the stretch mode ν(CC-O), 852 cm^−1^ to the stretch mode ν(C-CO) and 642 cm^−1^ to the wagging and twisting mode τ/*w*(O–H), and the creation of new bands at 1125 cm^−1^ and 990 cm^−1^ assignable to the stretch mode ν(Ti-OC) can be observed. It is very interesting to observe that the organic group survives hydrolysis and the formation of the inorganic scaffold by creating the Ti-O-Ti bridges. Figure 1c shows a scanning electron microscopy (SEM) micrograph of a TiBTO sample pointing to a typically laminar morphology. This fully matches the TiBTO XRD diffractogram (Figure 1d). Although the product is an amorphous solid, we observe a low-angle, wide reflection, centered at 2θ = 6.18°, similar to reflections assigned to 00l planes in laminar structures. In this case, we assign this characteristic to the reflection (001) of a layered arrangement with a repetition distance of about 14.3 Å.

The thermal analysis of the TiBTO (Figure S2) mainly shows three features, an endothermic peak at 102.6 °C with a loss in mass of ~17%, and two exothermic peaks with losses in mass of ~8% at 222.4 °C and ~18% at 416.4 °C, respectively. The endothermic character of the first peak is possibly due to both water evaporation and the increasing structural disordering of the host in the interlaminar space. The exothermic character of the observed peaks, weak at 222.4 °C and strong at 416.4 °C, would correspond—the former to the energy balance between the evaporation of the guest (b.p. 131 °C) and the formation of Ti-OH and/or Ti-O-Ti bonds involved in the formation of 3D-TiO_2_ that reaches its maximum speed at the second temperature.

#### 2.2.2. Titanium Oxide–Palmitic Acid (TiPA-A) via a Sequential Process

**Scheme 2:**
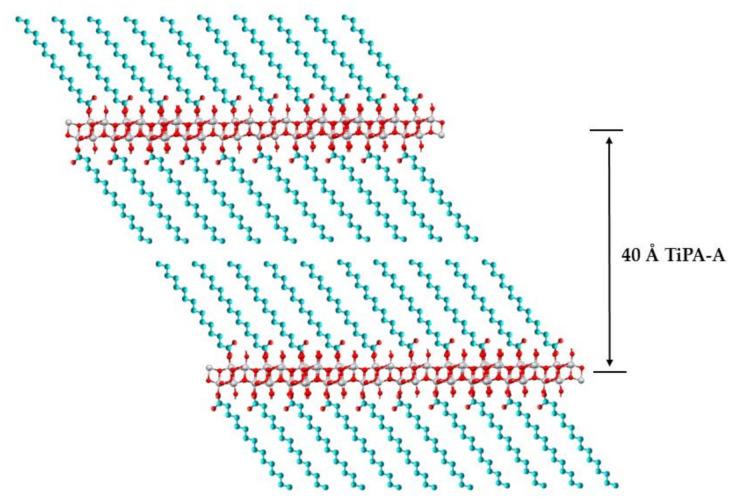
Suggested structural diagram for ideal bilayer structure of TiO_2_–palmitic-acid nanocomposite prepared by the sequential process, Type-A.

**Figure 2:**
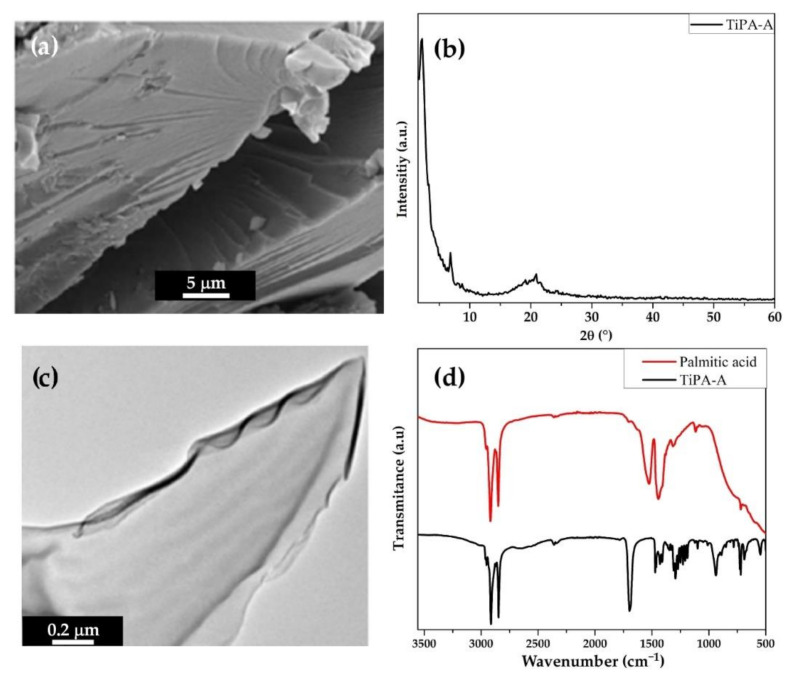
SEM (**a**), XRD diffractogram (**b**) and HTREM (**c**) of TiPA–A; and comparison of FTIR spectra of TiPA–A (red) and free palmitic acid (black) (**d**).

To evaluate the incidence of Brønsted acidity and the size of the incoming organic residue in the functionalization of titania with long-chain alkyl residues, we prepared a nanocomposite of TiO_2_ with hexadecanoic acid (palmitic acid, PA), labeled as TiPA-A, using a procedure similar to that used for TiBTO. Figure 2a–c shows scanning electron microscopy (SEM), transmission (TEM) images, and the powder X-ray diffraction (XRD) analysis of TiPA-A, respectively. The product has a laminar morphology made up of stacking very thin sheets, which agrees well with its diffractogram. Probably due to the effect of the chain of the organic precursor, TiPA-A exhibits a markedly higher crystallinity than TiBTO, allowing the detection of Bragg reflections 00l up to the order of three, at 2θ 2.18°, 3.30°, and 6.82°, respectively, with a repetition distance of 40.5 Å. The better stacking of the inorganic TiO_2_ sheets reflects a template hydrolysis process, enabled by a certain degree of self-assembly of the carboxylate-based hybrid surfactant produced by the carboxylic acid metathesis with TTIP in the first step of this method. However, the composition of the product, TiO_2_(PA) _0.72_·1.43H_2_O, (Table S1), shows that the insertion rate of the organic component is somewhat poor. This denotes that the higher acidity of the substituent was insufficient to overcome the effect of the viscosity of the reaction medium, which appears to be a major flaw of the sequential method. However, the compositional and XRD data, particularly the large intermolecular distance with respect to the molecular length of PA (~21.1 Å) and the projectable composition to a nanocomposite with a TiO_2_/substituent stoichiometry close to 1:1, suggest the formation of a bilayer structure of the type illustrated in Scheme 2.

Additional structural details are further discussed in Section 3. The FTIR spectrum of TiPA-A (Figure 2d) shows the typical absorptions of the alkyl residues of saturated fatty acids. However, there are also differences that indicate esterification by binding to the TiO_2_ matrix. Among them, the acyl ν(C=O) mode was observed at 1538 cm^−1^ in TiPA-A instead of the band at 1702 cm^−1^ for the same mode in the palmitic acid, which indicates the conversion of the acid into its carboxylate. Moreover, TiPA-A presents a new band assignable to the Ti-O-C bond. Although pristine PA has some water detected by the absorption mode δ(HOH) at 1630 cm^−1^ and a weak broad hydrogen-bond band centered at ~3442 cm^−1^, this was not practically observed for TiPA-A. Therefore, the small broad band centered at 3454 cm^−1^ mainly came from TiO-H bonds saturating sites not occupied by palmitate. Differential thermal analysis of the product (Figure S3) shows three exothermic peaks, at 346.8, 427.56, and 494.47 °C, associated with mass losses of 32%, 12%, and 14%, respectively. A mass loss of ~5% in the range of 80–180 °C of slightly exothermic character seems to be associated with structural water and dihydroxylation processes. The main loss of palmitates occurred between 230 and 440 °C. An intense exothermic characteristic centered at approximately 496 °C was mainly caused by the transformation of the mesostructured TiO_2_ into anatase and a small fraction of rutile. The latter, as well as the fact that the nanocomposite preserves its structure up to approximately 180 °C, is evidenced in the evolution of the XRD diffractogram of the product with the temperature shown in Figure S4.

### 2.3. The Concurrent Process

**Scheme 3:**
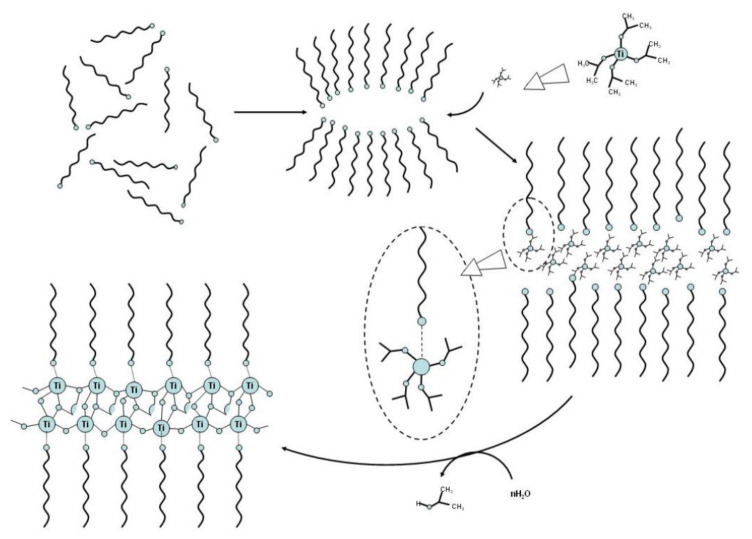
Concurrent process for the preparation of TiO_2_–fatty-acid nanocomposites type B.

This approach mainly differs from the sequential one in the temporality in which the reactions, metathesis, and hydrolysis can occur, as well as in that the process is carried out in a liquid medium. Scheme 3 illustrates the suggested process for the formation of TiO_2_–fatty acid nanocomposites by this method. The relatively slow rate of the metathesis reaction and the selected operating conditions allow both double decomposition and hydrolysis to occur, at least partially, almost simultaneously. In a one-pot process, the amphiphilic is dissolved in a low-polarity anhydrous solvent under conditions of suitable concentration, temperature, and standing time to stabilize a reverse micellar arrangement (Scheme S1). Then, after adding the TTIP dropwise, the reactor is open to the atmosphere, allowing the mixture to stand for 72 h. The concurrent strategy tested for the preparation of the nanocomposites with myristic (tetradecanoic, acid, MA), palmitic (hexadecanoic acid, PA), and stearic (octadecanoic acid, SA) fatty acids (FAs) proved to be quite adequate for incorporating long-chain alkyl derivatives into TiO_2_ in quantities even higher than the stoichiometric one, but generated products which were structurally different from those obtained by the sequential method. 

**Figure 3:**
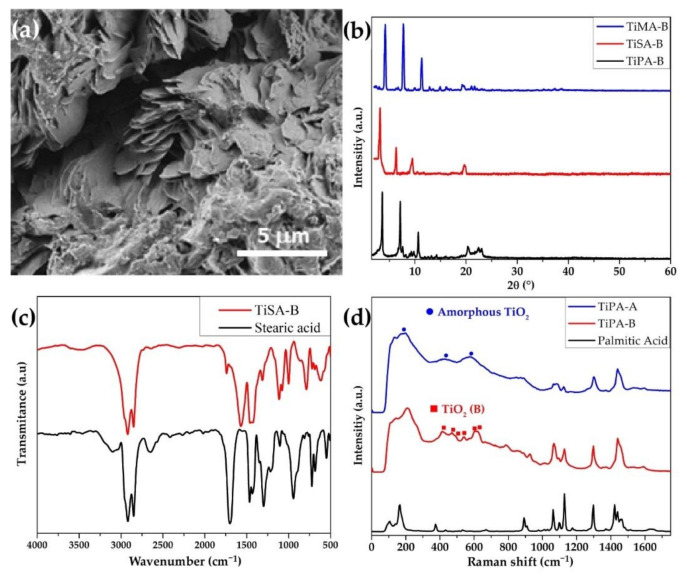
SEM micrograph (**a**) and XRD diffractogram TiO_2_–fatty acid (**b**); FTIR spectra of TiSA–B and stearic acid (**c**); and Raman spectra of TiO_2_–palmitic acid nanocomposites, prepared by sequential (TiPA–A) and concurrent (TiPA–B) methods, and free palmitic acid (**d**).

**Scheme 4:**
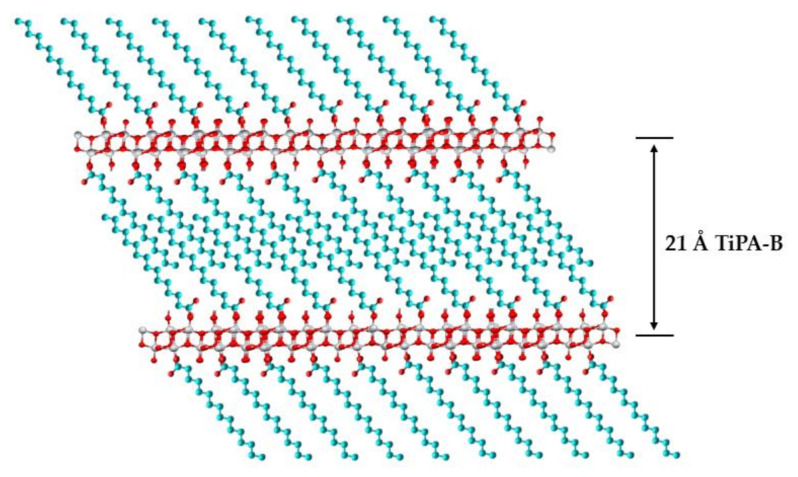
Suggested diagram for the interdigitated monolayer structure of TiO_2_–fatty-acid nanocomposites prepared by concurrent process, TiFAs-B.

Figure 3a,b exemplify SEM micrographs and XRD diffractograms, respectively, for the TiO_2_–fatty acid nanocomposites obtained by the concurrent method. Typical features of layered solids were visible in both SEM and XRD. The images showing micrometer particles with stepped surfaces and XRD profiles with one series of low-angle Bragg 00l reflections—with the repetition distance increasing with the molecular length of the organic guest, typical of laminar solids—are roughly similar to those of products prepared by the sequential method. However, the interlaminar distance in the products obtained using a solvent is comparatively much shorter, and the crystallinity and structural ordering of the sheets of the products prepared by the concurrent approach are clearly greater, where even the detection of a quantity of 00l reflection is sufficient to accurately assess their interlaminar distance. Furthermore, as observed in Table S2, the chemical composition data were obtained by elemental analysis complemented by thermogravimetric (TG) measurements for the three TiO_2_–fatty-acid nanocomposites prepared by the concurrent method, generically labeled as TiFAs-B. All these nanocomposites formally present a stoichiometric ratio Ti:FA of 1:1, though small deviations attributable to excess FA and/or different water content are often detected. Therefore, we suggest that TiFAs-B products belong to a family of nanocomposites with common properties that are generally different from those already described for TiPA-A. Relatively small interlayer distances, slightly less than the molecular lengths of the corresponding FAs, and the nearly equimolar stoichiometry of TiO_2_–FAs-B nanocomposites suggest monolayer structures of the type illustrated in Scheme 4. XRD diffractograms of some TiFAs-B samples show a low-intensity reflection at 2θ of about 20°, a region where no Bragg refraction for any of known TiO_2_ polymorph has been reported. This feature agrees with the reflections assigned to the lateral order of close packing alkyl chains in carboxylate/acids interspersed in layered double hydroxides (LDH) [31]. This is an important issue for describing the structure of TiFAs-B discussed later (Section 3). Further valuable information on the structural aspects of TiFAs arises from the FTIR and Raman vibrational properties of the products.

A representative example of the FTIR spectra of the TiFAs-B is presented in Figure S5. We comment on the FTIR spectrum of TiSA-B compared with that of pristine SA (Figure 3c). Most adsorption bands associated with alkyl residues in the nanocomposites appear at approximately the same positions as in SA. However, differences associated with the formation of the carboxylate similar to those commented for the TiPA-A above are also evident.

Figure 3d compares the Raman spectra of the TiPA samples prepared by the sequential (TiPA-A) and concurrent method (TiPA-B), respectively, with the spectrum of free palmitic acid in the range of 0–1750 cm^−1^. Characteristic Raman scattering of linear saturated fatty acids at around 1100 cm^−1^, 1300 cm^−1^, and in the range of 1500–1400 cm^−1^, assigned to modes ν(CC), CH_2_ torsion, and CH_2_ scissor vibrations and bending CH_3_, respectively [32], appear in approximately the same position in the spectra of the nanocomposites. However, the effect of incorporation on the fatty acids in the nanocomposite is often detectable by changes in the shape and relative intensity of the Raman peaks [33]. The most striking alterations in the spectrum of the alkyl moiety in organic fatty acids in nanocomposites with respect to pristine acids generally respond to trans-trans/gauche conformational disorders in alkyl chains. In fact, in systems containing linear alkanes, the increase in gauche conformers generally accompanies the transition from crystalline phases to more-fluid and less-ordered phases [34,35]. Some order/disorder features detected in Raman spectra of TiFAs-B are briefly discussed next for TiMA-B.

The relationship between the area (S) under the peaks belonging to the antisymmetric bending mode δ(CH_3_) and the scissor mode *sc*(CH_2_) that dominate the Raman scattering band centered at ~1440 cm^−1^ (Figure S6), [S δ_a_(CH_3_)]/[S sc(CH_2_)], is frequently considered as an indicator of the degree of uncoupling of the alkane chain. An increase in this ratio would reflect larger populations of free methyl and methylene groups capable of undergoing intramolecular movement [36]. Consequently, for the value of said indicator, for example, 1.16 and 1.06 for MA and TiO_2_–MA, respectively, the conformational order of the alkane chains in the nanocomposite is greater than in its precursor. On the other hand, chain–chain interactions also appear superior in TiO_2_–MA with respect to MA when comparing their respective intensity relationships between the methylene peaks, ν_s_(CH_2_) and ν_a_(CH_2_), at 2877 and 2847 cm^−1^, respectively. The decrease in [I ν_a_(CH_2_)/I ν_s_(CH_2_)], mainly related to the lateral packing density of the alkane chain [37,38], also occurred due to interdigitation between opposite chains [39], which agrees with the suggested structure for TiO_2_–FAs (Scheme 4) discussed in the next section.

The Raman spectra of the TiO_2_–fatty acids (B) already covered also provide valuable information on the structures of the inorganic moiety of the products. As previously reported [16,40], the sheets of titania in fatty acid composites correspond to the metastable polymorph of titania called TiO_2_ (B), which does not crystallize spontaneously, but uses an appropriate template or precursors under relatively soft conditions [41]. Therefore, the similarity of the Raman spectra of TiPA-B prepared by the concurrent method with TiO_2_ nanostructures, such as those reported for nanotubes [42], nanowires, or nanosheets [43,44], is not surprising. Some of the typical bands assigned to TiO_2_ (B) are consigned in the spectra in Figure 3d. It is worth highlighting the effect of the preparation method used in the preparation on the structure of the TiO_2_ layer in the nanocomposites described here [45]. The Raman spectrum of TiPA-A differs from those of the three TiPA-B, resembling that of amorphous anatase [46]. Figure 3d compares the Raman spectra of TiO_2_ nanocomposites with palmitic acid, prepared by the sequential (TiPA-A) and concurrent method (TiPA-B), respectively, with the spectrum of free palmitic acid in the range of 0–1750 cm^−1^. Figure S7 shows the same spectra as Figure 3d but focuses on the characteristic spectral range of the TiO_2_ polymorphs (100 and 800 cm^−1^) and includes that for the nanocomposite with 3-butynol, TiBTO. Without considering the palmitic acid peaks in that region (in 100–800 mm^−1^), the shape of the spectrum of TiPA-A that is similar to that of BTO but different from both TiPA-B and the phases reported for the most common types of TiO_2_ (Anatase, Rutile, Brookite and TiO_2_ (B) [42,44] can be tentatively classified as an amorphous phase. Meanwhile, the shape of the spectrum of TiPA-B, similar to that of nanocomposites with stearic or myristic acid prepared by the same method, suggests a structure close to that of TiO_2_ (B). This is consistent with reports of other TiO_2_ composites, organic–inorganic or with graphene [45], as well as the use of organic templates for the growth of TiO_2_ (B) [47].

#### 2.3.1. Photophysical Properties

**Figure 4:**
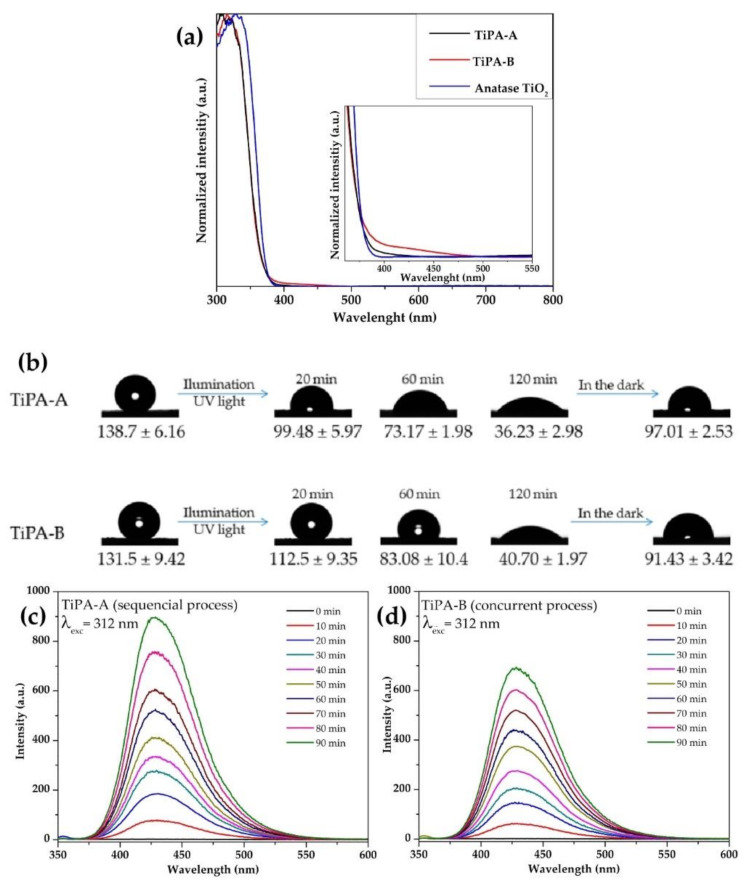
Diffuse reflectance spectra (DRS) of nanocomposites (**a**); contact angle measurements under dark and illumination at 365 nm (**b**); ^•^OH-trapping photoluminescence spectra of TiPA–A and TiPA–B in a solution of terephthalic acid at room temperature (**c**) and (**d**), respectively.

Figure 4a compares the diffuse reflectance spectra (DRS) of nanocomposites with palmitic acid prepared by the two methods discussed above with that of anatase, while Figure S8a,b show the absorption spectra of the three TiO_2_–FAs, as well as the evaluation of their bandgap using Tauc plots [48,49]. The results clearly show the semiconductor nature of the nanocomposites. The spectra are generally similar to those of anatase but reveal an increase in the separation band. Since the interleaving space increases slightly as the length of the alkyl chain increases, such an increase could be due to the confinement of TiO_2_ in a two-dimensional space [40]. However, other causes cannot be ruled out, such as the inductive effects of the organic substituent, since we observe, for example, that the bandgap for the 3-butyn-ol derivative is even higher (3.55 eV). Furthermore, as recently reported, [50] TiO_2_(B) grown on a thin-layer VO_2_-coated anatase would have a 3.6 eV bandgap, considerably higher than nanostructured anatase. In any case, the optical behavior of the TiO_2_ polymorphs remains a challenge because it is strongly dependent on the TiO_2_ polymorphs’ environment. In fact, the TiO_2_(B) found in nanotubes [51] or nanowires [52] ranges from 3.07 to 3.204 eV.

Although the TiO_2_–FAs samples show similar optical activity to TiO_2_, they also show some peculiarities not seen in bulk TiO_2_. For example, an almost-imperceptible feature at the red end of its absorption edge [53] possibly causes the frequent yellowing of the samples (Figure S8a), as well as its enhanced light-driven photocatalytic activity, which is discussed in the next section. The latter is despite the apparently detrimental aspects of these materials, such as their comparatively higher bandgap that makes it difficult to capture lower-energy light and the presence of an alkyl organic component that could be inconvenient for their interaction with the aqueous medium. To better understand the photoactivity of these materials, we made simple measurements of the surface tension of the particles and their sensitivity to the effect of light, as shown in Figure 4b. The contact angle of a drop of water with the surface of the particles of two samples of TiO_2_–PA without and with irradiation with an LED light source (WLS-22-A, Mightex) of 365 nm wavelength and 5 mW/cm^2^ power was studied. Illumination produced a marked increase in the hydrophilicity of the surface of both samples. The PL spectrum shown in Figure 4c,d corresponds to the formation of the highly fluorescent 2-hydroxyterephthalic acid radical by the oxidation of terephthalic acid with ^•^OH, which indicates the production of ^•^OH during the photocatalytic process of TiPA-A and TiPA-B [54].

#### 2.3.2. Photocatalytic Properties

**Figure 5:**
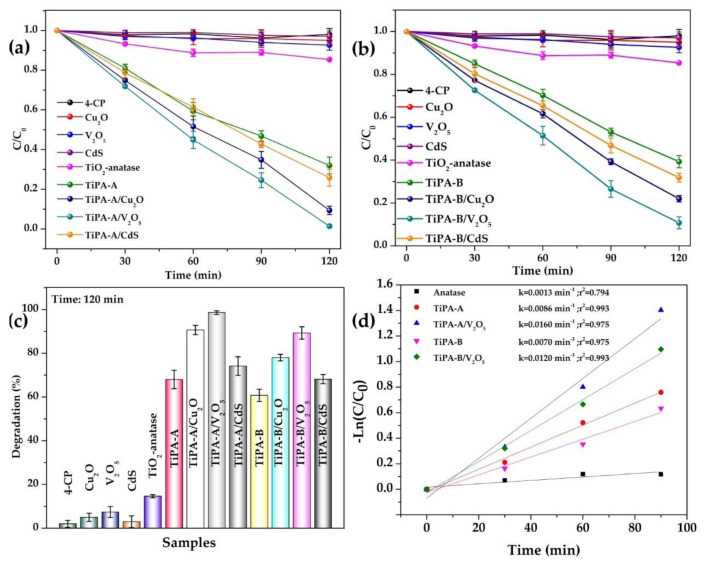
Photocatalytic degradation 4–CP 5 × 10^–5^ mol L^–1^ under simulated solar light irradiation assisted by TiPA and their composites with Cu_2_O, V_2_O_5_, and CdS (Controls: 4–CP and TiO_2_–anatase): Performance (C/C_0_) of TiPA–A (**a**) and TiPA–B (**b**), based photocatalysts. Rate of photodegradation (%) after 120 min irradiation, assisted by TiPA–A and TiPA–B, alone and by their composites with sensitizers (**c**). Kinetics of photodegradation assisted by TiPA–A and TiPA–B and their composites with V_2_O_5_ (**d**).

To complement the study of the synthesis, structure, and properties of supramolecular hybrid TiO_2_–FAs materials, we tested their ability to be adapted for solar energy conversion. The photocatalytic activity of TiPA-A and TiPA-B alone, as well as sensitized with lower-bandgap nanostructured semiconductors such as Cu_2_O, CdS, and V_2_O_5_ with direct bandgaps of 2.37, 2.36, and 2.40 eV, respectively (Figure S9), was validated by evaluating their efficiencies in the degradation of 4-chlorophenol (4-CP) as a pollutant model under simulated sunlight irradiation. As shown in Figure 5a,b, in all cases, synergy between the photoactivity of TiO_2_–PA and that of the sensitizers was observed.

Experiments were performed following the usual protocols in the current literature [16,55]. The photocatalytic response of both Ti-PA nanocomposites and their sensitized compounds was systematically compared with that of TiO_2_ anatase nanoparticles commonly used as reference photocatalysts. The experiment illustrated in Figure S10 shows the photodegradation of 4-chlorophenol assisted by the reference catalysts TiPA-A and TiPA-A sensitized with Cu_2_O nanoparticles indicated as TiO_2_–PA/sensitizers. Figure 5a–d compiles the photocatalytic performance in the simulated sunlight-driven degradation rate of 4-CP in terms of the degradation rate attained using each catalyst of the set of tested materials. All of them display a better performance than pure anatase, and the process rate constants involving TiPA-A were always slightly higher than those of TiPA-B alone, as well as with their respective composites, regardless of the nature of the sensitizing component. Complete photodegradation of 4-CP catalyzed by TiPA-A/V_2_O_5_ was achieved after 120 min with a rate constant of 0.016 min^−1^, approximately 2 times faster than TiPA-A and 12 times faster than anatase, as suggested by the graph ln (C/C_0_)/irradiation time, a regular degradation pseudo-first-order process that fits the Langmuir–Hinshelwood model (Figure 5d). The improved photocatalytic behavior of TiPA and TiPA-NPs heterosupramolecular materials with respect to anatase agrees with previous reports on other TiO_2_-based systems using other fatty acids and different pollutants targets [16,55] and reinforces the suggested trend of the concept that soft hybrid semiconductors could be an alternative to conventional purely inorganic photocatalysts. Due to their large bandgap, the best photocatalytic performance of the TiO_2_–PA-NPs samples could be attributed to the decrease in the recombination of the photogenerated electrons and holes and a closer interface between the heterosupramolecular materials with the surfaces of the nanoparticles.

## 3. Discussion 

Manufacturing stable laminar species from inherently three-dimensional inorganic compounds is an ever-interesting challenge. TiO_2_, like many metal oxides in its standard state, crystallizes as three-dimensionally extended, strong, covalent, ionic metal–oxygen bond networks. Obtaining stable single sheets of this type of material implies saturating the pendant bonds through thermodynamically strong interactions with their environment, which necessarily results in chemical species other than pristine oxides. Therefore, using a nomenclature based on any of the pure TiO_2_ polymorphs to identify materials such as those described here is a crude approach. However, this approximation, based on the similarity of specific physicochemical properties—in our case, its behavior as a semiconductor—although unacceptable in conventional chemistry, is currently understandable and often necessary in the field of nanochemistry. Given the nature of the structural unit of our products, a molecular layer of TiO_2_ stabilized and protected by a self-assembled monolayer of carboxylate ions is needed; we have generically described them as core–shell structures, TiO_2_–surfactant, using the commonly applied term for coated nanoparticles that are generally spherical. The successful stabilization/protection of the core of TiO_2_–surfactant nanocomposites and their behavior as quantum material in bulk products is evidenced in their thermal stability, often up to more than 100 °C under ambient conditions (Figure S4), and in the decrease in their bandgap relative to bulk TiO_2_ (Figure S8b), respectively. All the products were prepared from the same precursors and similar reaction conditions, but using two slightly different procedures, which we call the simultaneous process (A) (Scheme 1) and concurrent process (B) (Scheme 3), respectively, depending on the moment in which the reactions, double displacement, and hydrolysis, involved in the synthesis preferably occur.

Both methods A and B generate products of the same type, TiO_2_–fatty acid. However, as often occurs in supramolecular or nanochemical processes, changes in subtle variables such as the temporal concentration of the reagents and the mobility of the reagents and intermediates in the medium throughout the process can alter the characteristics of the final products. Thus, significant differences in the yield and stoichiometric deviations of the products, in their core and shell molecular structure, and in the architecture of the nanomaterials are apparent.

The separation of the intermediary [Ti(TIP)_3_(BTO)(OH)_2_]_2_O (mp-TiBTO) in the preparation TiO_2_–BTO via method A teaches us about the stoichiometry and the sequence of the metathesis and hydrolysis reactions involved in the syntheses and the concurrence between both processes. Traces of water favorable for promoting the metathesis [56] were enough to simultaneously induce partial hydrolysis, leading to the molecular dimer mp-TiBTO (Figure 1b) instead of the monomer. As observed in Table S1, the same effect is observed for the nanocomposites TiBTO and TiPA-A. The inclusion rate of the organic component into the inorganic matrix afforded by this method appears relatively poor due to the formation of byproducts (Table S1), denoting incomplete precursor metathesis in the first stage of the procedure (Scheme 1) and/or deficient aggregation-drive self-protection of a hybrid amphiphilic intermediary along the hydrolysis stage. Since, in the TiPA-A nanocomposite, part of the non-ionized carboxylic acid is not detected by vibrational analysis or XRD, it seems to be co-intercalated in the samples, although it maintains the structure of the product. Therefore, at least from a compositional point of view, it is not a properly commensurate nanocomposite. The relatively high viscosity of the medium due to the use of neat precursors appears to be a major flaw of the sequential method. Contrastingly, concurrent method B, using a solvent and maintaining the amphiphilic to TTIP ratio higher than one during the reaction, allows the surfactant to self-assemble for templating both metathesis and hydrolysis processes, avoiding intermediary hydrolysis hybrids. In this case, the products have comparatively higher crystallinity, and the impurities mainly consist of the excessive incorporation of fatty acid. The fact that the stearic acid is nearly stoichiometric reveals the importance of the amphiphilic properties of the precursor.

Regarding synthesis strategies’ effects on the structure of products, the results indicate that the inorganic core in TiPA-A is structurally similar to anatase, while in TiPA-B, as well as in the myristic and stearic derivatives prepared by the same method, the structure of the core resembles the TiO_2_(B) polymorph. Moreover, the carboxylate chains in the TiPA-A shell have a conformation all-trans similar to that of form C saturated fatty acids under normal conditions [32], while the carboxylate chain in TiFAs-B corresponds to the B form of the pristine acids, characterized by a gauche conformer between the C2-C3 carbons of the alkyl chain [57]. In relation to the supramolecular architecture of bulk nanocomposites, the most relevant and easily identifiable difference between TiPA-A and all TiFA-B nanocomposites is the interlaminar distance (**d**). In the former, **d** is close to twice the length of the palmitic acid molecule, while in TiFAs-B, **d** is a bit shorter than the corresponding surfactant length. Since in both cases the amount of FA is the same, both TiO_2_–FA nanocomposites correspond to two different polytypes, differing in the manner that the single hybrids are stacked, leading to an organic interphase constituted either by a tail-to-tail bilayer (Scheme 2) or by a monolayer of interdigitating surfactant molecules (Scheme 4). There is some similarity with free fatty acids, for example, with the polytypes Mon and Orth of the polymorph B of stearic acid [58], where acid molecules arrange in bilayer or monolayer sheets, respectively. However, the polytypes of natural saturated fatty acids free or in lipids are always bilayer arrangements, except in some choline phospholipids where interdigitating structures are detected [59]. However, the interdigitated monolayer architectures are well known and easy to achieve in synthetic systems that, as discussed later, are in some respects quite similar to ours [60].

The preparation of the TiO_2_–PA nanocomposites is a striking example of polymorphic conversion caused by subtle energetic changes shows the potential of supramolecular chemistry to modulate the properties of hybrid materials. A reaction medium where the mobility of the reagents is poor, as in method A (pure reagents with relatively high density) (Scheme 1 and Scheme S1a), is unfavorable for the surfactant’s self-assembly. Under such conditions, the metathesis of precursors creates disordered amphiphilic hybrids, which may cause partial hydrolysis to byproducts before giving rise to core–shell products, where both parts correspond to the most stable polymorphs of the precursors, anatase-type core, and carboxylate shell species in form C fatty acids. On the other hand, method B, where the mobility of the precursors (solvent) is greater (Scheme 3 and Scheme S1b), favors the self-assembly of surfactants, which promotes an uphill polymorphic transformation process, creating core–shell nanocomposites where both components, TiO_2_(B) and fatty acid form B, are thermodynamically metastable with respect to their parent compounds.

There are many intrinsically layered inorganic ion exchangers, such as layered natural clays [61] or layered double-metal hydroxides (LDHs) [62], where interleaved monolayers or a bilayer of interleaved long-chain surfactants can be easily obtained by intercalation driven by ion exchange when using guest ion concentrations appropriately greater than the host ion’s exchange capacity (CEC) [63]. Thermal conversion of bilayer architecture to a monolayer has also been reported in some carboxylates intercalated with LDHs [64]. This agrees with reported phase-transition temperature measurements for a phosphocholine lipid derivative demonstrating that interdigitating is thermodynamically less favorable than the formation of bilayers [59]. That notwithstanding, the tendency of LDHs to incorporate neutral carboxylic acid molecules beyond the CEC to fill the voids in the matrix surface is spontaneous due to the metastability of the stoichiometric product by packaging frustration with respect to close packing corresponding to the critical parameter of packing (CPP), close to one of the extended saturated fatty acids [65,66]. The behavior of the LDH/fatty acid composites helps us to better understand the origin of the structural versatility that we observe in the TiO_2_–FA nanocomposites. Both excessive guest absorption with respect to the CEC of the host and chain-interdigitating packing would essentially have two tasks, keeping the CPP [67,68] of the pristine fatty acid close to one and laterally compressing the parallel chains until the distance between interfaces is optimal to maximize the stabilizing effect of the van der Waals forces (VdW). The resulting exergonic process is a determining factor for the structure that fatty acids acquire in the interleaving space. Since the CPP of fatty acid-like surfactants mainly depends on both the volume of the hydrophobic tail and the effective area of its head, we suggest that the effective head area in commensurate lamellar hybrid compounds would be equivalent to the distance between the sites of anchoring of two adjacent methylene chains. Although this distance is a fixed parameter that subtracts one degree of freedom from the adjustment of the critical packing parameter, this is compensated for by the variability of the angle defined by the direction of the linear tails and the normal host basal plane. This is observed in biological systems, where the variation of this angle provides a fine adjustment tool, sensitive to the interaction with the medium, to minimize the system’s energy while maintaining the optimal distance between the hydrocarbon chains. For example, in pure natural fatty acids and lipids, the hydrocarbon tails are arranged in parallel and generally inclined with respect to the normal plane formed by hydrogen bonds between their acyl groups or the molecules to which they are esterified. The energy minimum corresponds to a compromise between the structural and/or steric energy cost of the angle adaptation and the optimization of the VdW energy. Regarding the lateral compression of chains, this is necessary to achieve the proper distance of each system, which maximizes the attractive interactions between the chains.

Because VdW forces are non-specific and attractive short-range interactions based on the Lennard–Jones potential, they do not leave much room for differences between the optimal distances for similar chemical systems. In fact, XRD measurements for semisolid samples of a series of fatty acid methyl esters allowed the definition of crystalline sub-cells whose shorter dimensions corresponded to the distance between two neighboring chains (equivalent to the VdW diameter), which are in the range of 4.59–4.55 Å [69]. On the other hand, the reported lateral Bragg refractions for intercalated stearate ion samples in different LDH matrices point to VdW diameters of 4.10 and 4.51 Å [63], at room temperature, or changing from 4.06 Å to 4.25 Å by heating above 85 °C [31]. In all these cases, the products present the characteristic all-trans conformation of the C-form of fatty acids. For our part, the XRD measurements of a TiO_2_–SA-B sample, which, as mentioned above, presents the gauche conformation characteristic of the B form of fatty acids, reveals distances of ~4.51 Å, which, we suggest, correspond to the separation between two contiguous carboxylate chains. This value is somewhat higher than previously considered for the LDH compound [31,63]. However, this difference is consistent with those observed between the shorter sides of the crystal unit cell of form C and form B of pure stearic acid [70].

The precise structural description of hybrid-layered products is still a big challenge. In laminar structures such as LDHs or ours, crystallographic coherence is limited to the stacking direction of the lamellae due to turbostratic disorder caused by the lack of strong interactions between the sheets. Therefore, XRD data are generally limited to determining the distance between the inorganic layers. The approach is generally used to describe the structure of lamellar clay, and LDH-based hybrid composites consist of simple models based on fitting the ratio of the cross-sectional area of the methylene chain to the available area in the inorganic matrix [54]. Since the experimental interlaminar distances (**d_XRD_**) are always shorter than those expected for linearly extended fatty acid carboxylate chains, the latter must be inclined, defining an angle of inclination (**α**) with respect to the normal basal plane of the lamina [71]. Therefore, describing the structure of the interleave phase within the frame of this model implies knowing the length of the supposed linear methylene chains. The optimization of lateral chains interactions assumes that the linearity of n-alkyl carboxylate molecules required for these models is quite reasonable. However, their real chain molecular dimensions are more difficult to assess due to the conformation molecules adopt under confinement conditions. In the case of families of composites where alkyl chains present an all-trans conformation, as generally occurs in LDH intercalates, these models satisfactorily reproduce experimental interlaminar distance and estimate the change deviation angle (**α**) by assuming the large size determined for all-trans n-alkanes (about 1.27 Å per CH_2_ group) [72]. Indeed, this feature agrees with the slope of the linear function of the XRD repeat distance on the number of chain carbon-atoms determined for a variety of linear fatty acids and similar surfactants [73]. Important limitations of the original LDH model concern said surfactant conformation and the assumption that extended chains entirely cross the interleaving space. Although the latter can be resolved by additional constant terms, for instance, by considering the space occupied by terminal methyl end-groups [74], such an approach is difficult to assess in the case of partial interdigitating and/or unknown contribution of a chain’s conformational changes, as is the case of TiFAs-B, where both a different FA conformation and partial interdigitating are apparent.

For analyzing the structure of our products, we employed the model illustrated in Scheme S2a,b, representing in a flat form the bilayer and monolayer nanocomposites TiO_2_–surfactant nanocomposites. These schemes are similar to those used for the LDH composites already mentioned, but consider the peculiar commensurateness of the TiO_2_-based products highlighted above, but implying that parameter **a**, the distance between two neighboring carboxylate anchor sites, for a given matrix is fixed. In our cases, the parameters **a** and **d_TiO2_**, the width of the inorganic layer, are determined by the crystal structure of the TiO_2_ sheet, so the free interlayer space, **d_free_**, can be directly calculated from the experimental XRD (00l) repeat distance (**d_XRD_**). Parameter **t**, introducing the minimal energy condition (VdW diameter of the chains), as discussed above, is typical of the system under given work conditions, and it is possible to estimate either experimentally from Bragg lateral reflections or from the literature [30,55,61]. For a family of products, the intensity of lateral reflections at ~20° in 2θ is generally very low and often impossible to detect, but their position is always the same independent of the surfactant. Precision in assessing all these parameters may improve with accurate measurements and/or using better-quality samples. However, optimizing **d_XRD_** and **t** in the same sample is generally tricky since the intensity of the 00l reflection increases with the number of sheets per particle, while that of lateral reflections depends on the basal area and the number of particles.

In these systems, α is defined by its cosine t/a and simultaneously by cos(**d′**/**L**). There, **L** is two or one times the molecular length of the surfactant, **l**, for a bilayer- (Scheme S2a) or monolayer (Scheme S2b)-type structure, respectively, complementing their corresponding lengths with **x** in the direction of **l** to reach the core surface of the opposite lamina. For bilayer arrays, **x** matches the spacing between opposite strands, while for single-layer structures, its subtraction from **l** corresponds to the length of the interdigitated zone **x_int_**. Although, as mentioned above, TiPA-A is an amorphous material and is not exactly a commensurate nanocomposite, we tested whether it responds to the proposed model. Assuming a core with an anatase-like crystal structure, a carboxylate tail length for an all-trans conformation, and a VdW diameter similar to those observed for LDH composites (4.06 Å) [31], we determined an inclinator angle of 49.3° and a tail-to-tail gap (**x**) of 4.9 Å. Despite the limitations of this system and the low reliability of the data, the results are quite acceptable. The tilt angle **α** is below the range for carboxylates or intercalated dodecyl sulfates intercalated in LDHs (60°–55°) [31,62]. The value of the methyl gap is not too far from the VdW diameter reported in the literature (5.6 Å) [72]. However, the question remains whether these results really correspond to a bilayer itself or an intermediate situation between it and a partially intercalated double layer with an excess of guests, as often occurs in non-commensurate compounds such as intercalated LDHs, which are generated spontaneously for energetic reasons. Clarifying this point is currently a pending challenge. The available data for TiFA-B nanocomposites are more reliable for testing the model due to the quality of the samples and the use of an experimentally determined **t**-parameter (4.51 Å), as well as the commensurability of the products. The parameter **a** was obtained from a slightly distorted structure of TiO_2_(B) that allows a distribution of carboxylates according to the stoichiometry of the nanocomposites, where the distance between two neighboring chains is 6.051 Å and the thickness of the TiO_2_ sheet is 4.8 Å. The results indicate a tilt angle **α** of 41.3° and a chain interdigitating of 27.7, 25.9, and 31.3% for TiO_2_–FA-B nanocomposites with MA, PA, and SA, respectively. In general, interdigitating appears to increase with chain length, but palmitic acid shows apparently anomalous behavior. This could be related to the spacing between adjacent methyl carboxylate molecules (short-range spacing), which, as determined by XRD measurements at temperatures near the melting points, would be 4.59, 4.62, and 4.55 Å for MA, PA, and SA, respectively [69]. Concerning the slight difference in the angle observed for TiPA-A with respect to the TiPA-B, as well as the fact that its value is significantly lower than those reported for the compounds with LDH, may be rationalized considering the commensurability of the TiFA-B nanocomposites. This is contrary to ion exchangers, where the anchoring sites determined by their respective CECs leave more space to accommodate the chains without forcing the natural inclination of the chains with respect to the normal direction of the sheets (90 − α). In the case of a bilayer structure of a commensurate TiPA-A, with a lower density of organic matter in the interleaving space, a tilt angle closer to that observed for interlacing based on LDHs would be expected. This is in agreement with the behavior of a TiPA-A intermediate between those corresponding to the LDH and TiO_2_ matrices already mentioned.

Despite the structural and compositional differences between products caused by different preparation methods, their photophysical properties are quite similar. This suggests that the architecture of the intercalated organic phase and even the polymorph of the inorganic moiety do not significantly affect the structure of the electronic structure of the materials, which beyond the confinement-induced alteration of the bandgap, appears to be qualitatively the same as that of pristine TiO_2_. On the other hand, the photocatalytic activity of the TiO_2_–FAs nanocomposites is clearly superior to that of anatase under the same conditions. Therefore, the key factor in photocatalytic activity appears to be the single-layer structure of the semiconductor, which, together with extended organic–inorganic interfaces, would favor the photocatalytic efficiency by lowering charge carrier recombination. Improved lower energy light absorption, as suggested by the slightly Urbach tail in the nanocomposite UV-vis spectra, would also be promoted. Moreover, since spectra do not indicate new stable inter-bandgap stable electron states, enhanced photoactivity in the visible region should also be associated with a large area of interfaces naturally rich in defects. In line with the latter is the fact that the photocatalytic activity of TiPA-A is slightly superior with respect to TiPA-B. The organic–inorganic interface in TiPA-A, as indicated by its composition, is chemically more disordered than that of TiPA-B because TiO_2_ sites seem to be saturated not only by carboxylate groups but also with non-ionized carboxylic acid molecules that are probably stabilizing the remaining Ti-OH groups. The results, in general, corroborate that, with regard to photocatalysis, the main advantage of this type of supramolecular entity is the 2D structuring of TiO_2_, where the phase purity of the materials is relatively less important, which is undoubtedly interesting from the point of view of possible applications.

Regarding the interface of the catalyst with its environment, the increase in the hydrophilicity of the particle surface increases when illuminated, indicating that charge transfer occurs from the core to the shell surface. Therefore, the photocatalytic effect on the contaminants in the solution, as well as with other semiconductors in the composites, can occur at a distance, so that in this case, intimate contact is not always essential to produce a charge transfer. Although there are still many open questions, including the real role of the surfactant layer beyond protecting the TiO_2_ core to achieve the performance of this type of hybrid photocatalysts, the results reported in this work can be seen as a proof-of-concept of the potential of hybrid semiconductors for the development of new and versatile supramolecular photocatalysts. Finally, it is worth mentioning that the photoactive TiO_2_–FA nanocomposites described in this work could be of interest for biomedical and cosmetic applications, for example, photodynamic and theragnostic therapy treatments based on TiO_2_ NPs [75], and the development of cosmetic formulations that combine TiO_2_ NPs’ beneficial role as a UV-blocking filter and block the toxicological risks of TiO_2_ NPs through the protective effect of fatty acids that reduce the penetration of NPs into cells [76].

## 4. Materials and Methods

### 4.1. Chemicals

All chemical reagents used in the experiments were obtained from commercial sources as guaranteed grade reagents. Titanium (IV) isopropoxide 97% (TTIP: Ti[OCH(CH_3_)2]_4_), 3-butyn-1-ol 97% (HC≡CCH_2_CH_2_OH or C_4_H_6_O), 1-octanol 98% (CH_3_(CH_2_)_7_OH), chloroform 99% (CHCl_3_), acetone ACS reagent ≥ 99.5% (CH_3_COCH_3_), ethanol absolute (CH_3_CH_2_OH), 4-chlorophenol (4-CP), Cu(CH_3_COO)_2_, ascorbic acid (C_6_H_8_O_6_), hydrazine (N_2_H_4_), hexadecylamine (HDA), vanadium triisopropoxide (VOTPP), cadmium chloride (CdCl_2_), sodium sulfide (Na_2_S), and terephthalic acid (TA) were purchased from Sigma-Aldrich (St. Louis, MO, USA). All reagents were of analytical grade and were used without further purification.

#### 4.1.1. Synthesis of Titanium Oxide–3-Butyn-1-Oxide (TiBTO)

The synthesis was carried out by condensing TTIP with 3-butyn-1-ol under an inert Ar atmosphere at room temperature. Totals of 10.0 mmol (2.93 g) of TTIP and 10.0 mmol (0.72 g) of 3-butyn-1-ol were mixed. The mixture was stirred with heating (70 °C) for 30 days. The purified partial product was obtained by distillation at 120 °C under 1 × 10^−3^ Pa. Then, it was exposed to the air and atmospheric humidity at room temperature to allow hydrolysis. After 24 h, it was milled and dried under vacuum (1 × 10^−3^ Pa). An amber solid was obtained as a final product after successive washes with acetone and dried at room temperature.

#### 4.1.2. Synthesis of Titanium Oxide–Palmitic Acid Sequential Approach (TiPA-A)

The synthesis was carried out by condensing TTIP with palmitic acid under an inert Ar atmosphere at room temperature. Totals of 10.0 mmol (2.93 g) of TTIP and 10.0 mmol (2.85 g) of palmitic acid were mixed. The mixture was stirred with heating (70 °C) for 10 days. The purified partial product was obtained by distillation at 120 °C under 1 × 10^−3^ Pa. Then, it was exposed to the air and atmospheric humidity at room temperature to allow hydrolysis. After 24 h, it was milled and dried under vacuum (1 × 10^−3^ Pa). A yellowish solid was obtained as a final product after successive washes with chloroform and acetone and dried at room temperature.

#### 4.1.3. Synthesis of Titanium Oxide–Palmitic Acid Concurrent Approach (TiPA-B)

During synthesis, 6.4 g of palmitic acid was mixed with 23 mL of ethanol in an argon atmosphere; the solution was stirred for 3 h at 50 °C. Then, 4.5 mL of TTIP was added dropwise, and the suspension was stirred for 1 h at constant temperature and repose for one week at 25 °C. The resulting white precipitate was separated by centrifugation, washed three times with ethanol, and dried at 45 °C for 72 h. A white solid was obtained as a final product.

#### 4.1.4. Cu_2_O Nanoparticles

A total of 20 mL of ethylene glycol and 0.38 g of Cu(CH_3_COO)_2_ were added at 40 °C under constant stirring in an argon atmosphere for 40 min. To the resulting solution, ascorbic acid (C_6_H_8_O_6_) and hydrazine (N_2_H_4_) were sequentially added. The solvothermal treatment (ST) was kept at 180 °C for three days under self-generated pressure. The nanoparticles were obtained using the same synthetic conditions as reported by Segovia et al. [77].

#### 4.1.5. V_2_O_5_ Nanotubes

A solution of 10^−3^ mol of HDA in pure ethanol, previously de-gassed, was mixed with 2 × 10^−3^ mol of VOTPP. The orange suspension obtained after stirring for 24 h was subjected to hydrothermal treatment in a Teflon-lined autoclave at 180 °C for 6 days. The nanoparticles were obtained using the same synthetic conditions as reported by Benavente et al. [78].

#### 4.1.6. CdS Nanoparticles

The nanoparticles were synthesized by a reported method in the literature, using cadmium chloride (CdCl_2_) and sodium sulfide (Na_2_S) taken as cadmium and sulfur sources, respectively [79].

#### 4.1.7. (TiO_2_–PA)/Nanoparticles Composites

The samples were mixed mechanically in an agate mortar (relation *w*/*w*). Before irradiation, the suspension was ultrasonicated for 2 min to produce homogeneous and non-agglomerated samples.

### 4.2. Characterization Methods

X-ray diffraction (XRD) analyses of the products were performed using a Bruker D8 Advance (Cu Kα λ = 1.5418 Å). The images of scanning electron microscopy (SEM) were obtained by using a Quanta FEG250 and EVO MA 10 ZEISS microscope, and the images of transmission electron microscopy (TEM) were obtained by using a JEOL JEM 2200FS (200 kV). The samples for TEM analysis were prepared by depositing and then drying them on a Cu grid, a slurry prepared by dispersing the product in ethanol. The diffuse reflectance spectra (DRS) in the range of 300–800 nm were performed using a Shimadzu double beam (model 2450 PC) spectrophotometer. Reflectance measurements were converted to absorption spectra using the Kubelka–Munk function. Thermogravimetric analyses were recorded in TG/DSC 1100 SF Mettler Toledo. Photo-luminescence spectra were recorder in a fluorescence spectrophotometer PerkinElmer (model LS 55). For photocatalysis, the absorption spectra of the samples were recorded at room temperature in a UV-vis spectrophotometer Jasco (model V-730). Raman spectra were obtained by a Raman Alpha 300 spectrometer, WITec GmbH, using an Nd:YAG laser (532 nm) for excitation. All the measurements were completed at room temperature. The contact angle was found using a ThetaLite optical tensiometer from Attension-Beloin Scientific (Gothenburg, Sweden).

### 4.3. Photocatalytic Activity and Hydroxyl Radicals

#### 4.3.1. Photocatalytic Degradation

The photocatalytic activities of the nanocomposites were studied for the degradation in visible light irradiation of 4-chlorophenol (4-CP) as a pollutant model. For this study, the characteristic absorption peaks of this compound, as an aqueous solution, located at 280 and 225 nm, were monitored using a UV–Vis spectrophotometer at regular intervals and the corresponding absorption spectra. 

The photocatalytic experiments were carried out by adding 10 mg of TiO_2_–PA with nanoparticles in 1:0.01 molar proportions in 25 mL of a 5 × 10^–5^ mol L^–1^ in solution at pH 7.0. Prior to the measurements, the suspension was kept in the dark for 30 min to reach adsorption/desorption equilibrium. The suspensions were irradiated by a solar simulator, VeraSol-2 LED Class AAA (Oriel^®^), using an intensity of 1000 W/m^2^ (1 sun). All samples had constant magnetic stirring to ensure a higher level of homogeneity of the photocatalyst in the suspension. Approximately 0.35 mL of the reaction mixture was taken out at different times and then centrifuged to avoid light scattering due to interference from the suspended ca8talyst particles. The 4-CP concentration after equilibration was regarded as the initial concentration (C_0_) and was monitored in the UV–Vis spectra of the solution using nanopure water as a reference. The degradation rate of phenol is expressed in terms of the relation: D = (Co − Ct)/Co × 100%.

#### 4.3.2. Hydroxyl Radicals

The production of hydroxyl radicals (^•^OH) was detected by photoluminescence (PL), in an aqueous suspension of the photocatalyst with terephthalic acid (TA) illuminated with simulated visible light. The experimental procedure was similar to the photocatalytic activity measurement, with a 4 mM TA solution in an alkaline medium (pH = 8). The tests were performed every 10 min, taking 5 mL of the solution to analyze the fluorescence, and then returning the aliquot to the suspension.

## 5. Conclusions

We studied lamellar organic–inorganic supramolecular hybrid nanocomposites’ core–shell type consisting of a 2D-TiO_2_ molecular layer stabilized on both sides by a self-assembled monolayer of carboxylates with a long alkyl chain through bottom-up processes consisting of the metathesis of the precursors TTIP and either fatty acids or 3-butinol, followed by the slow hydrolysis of the products. Two procedures were used that only differed in terms of the most likely instant of the metathesis/hydrolysis processes, sequential (method A) or concurrent (method B), both giving rise to similar products in terms of their equimolar formal Ti/surfactant, lamellar structure, and optical and photocatalytic properties, along with some interesting morphological differences. Among them are the titania core polymorph, similar to anatase or TiO_2_(B); the guest carboxylate conformers, either Form C or Form B of the fatty acid; the type of structure of the nanocomposite and arrangement of the alkyl chains, whether bilayer or monolayer, all of the above depending on whether the products are prepared by method A or B.

The most prominent feature of TiO_2_–surfactant structures, compared to known hybrid layer nanocomposites such as clay or layered double hydroxide-based ion exchangers, is their intrinsic commensuration. There, each TiO_2_ unit is a suitable site to anchor the surfactant, and the distance between the neighboring anchor points of the surfactant to the titania lattice has a fixed value which we suggest should mirror the effective diameter of the carboxylate head. This permitted us to estimate the ideal tilt angles of the acyl chains with respect to the basal plane of the sheets from experimental data sheets. Therefore, we use conventional geometric models that mainly consider critical packing parameters for micellar systems and energy minimization by optimizing van der Waals interactions to assess the degree of chain crosslinking in interdigitated monolayer systems or the space between the terminal methyl groups of opposing methylene chains into bilayer arrangements.

TiO_2_–surfactant nanocomposites have semiconductor properties regarding the conversion of solar energy into chemical energy both in their pure state and in composites with semiconductors with a lower bandgap used as sensitizers, improved with respect to nanostructured anatase under similar conditions. Such photocatalytic behavior of the photoactive products presented under solar illumination constitutes a proof-of-concept of the potentiality of hybrid semiconductors for developing new and versatile supramolecular photocatalysts. Furthermore, the results can be interesting for healthcare-related cosmetic applications.

## Data Availability

Not applicable.

## Supplementary Materials

The following are available online at https://www.mdpi.com/article/10.3390/ijms23074006/s1.
