# Supplementary material for: Two-Dimensional Titanium Dioxide–Surfactant Photoactive Supramolecular Networks: Synthesis, Properties, and Applications for the Conversion of Light Energy
Source: Int J Mol Sci. 2022 Apr 4;23(7):4006. doi: 10.3390/ijms23074006 (PMC8999612; doi:10.3390/ijms23074006)
Supplement: Supplementary file 1 [file ijms-23-04006-s001.zip › ijms-1613646-supplementary.pdf]

International Journal of *Molecular Sciences*

MDPI

Supplementary Information

Two-Dimensional Titanium Dioxide–Surfactant Photoactive Supramolecular Networks: Synthesis, Properties, and Applications for the Conversion of Light Energy

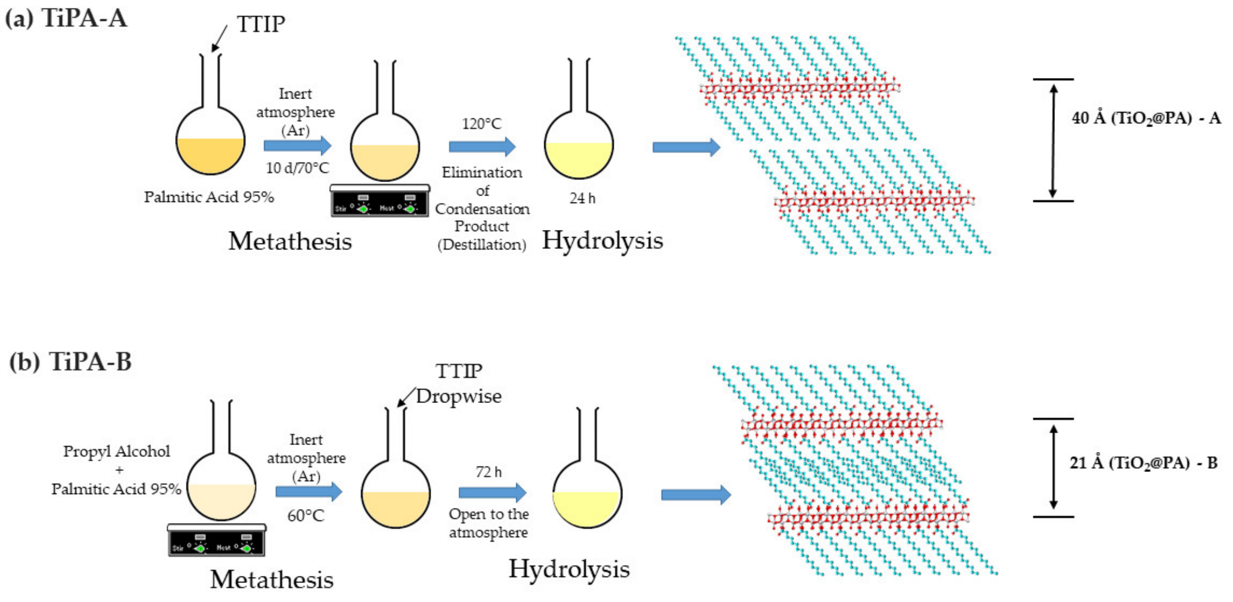

Scheme S1. Diagram of synthesis methods of TiO_2@Surfactants nanocomposites. Sequential process (**a**) and Concurrent process (**b**).

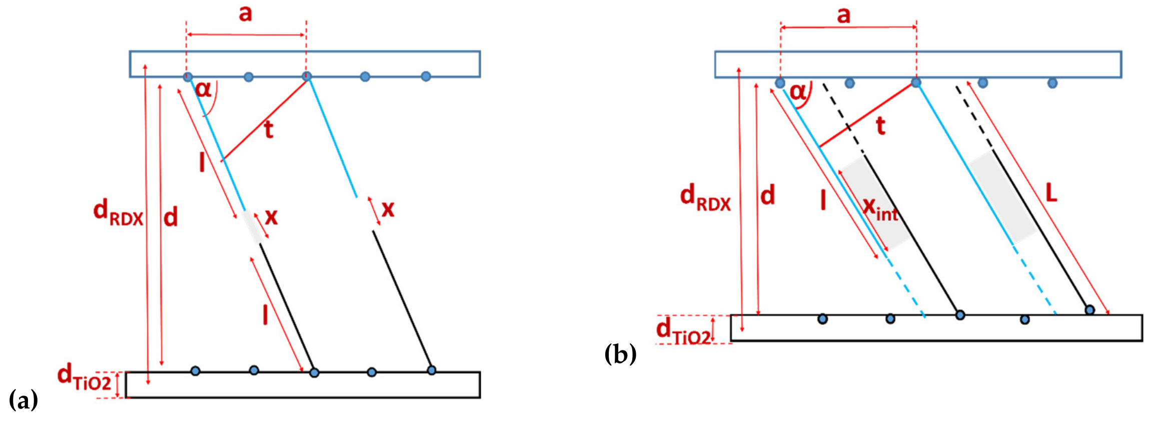

Scheme S2. Geometric model to describe the structure of commensurate TiO_2@fatty acids. Sequential process (**a**) and Concurrent process (**b**).

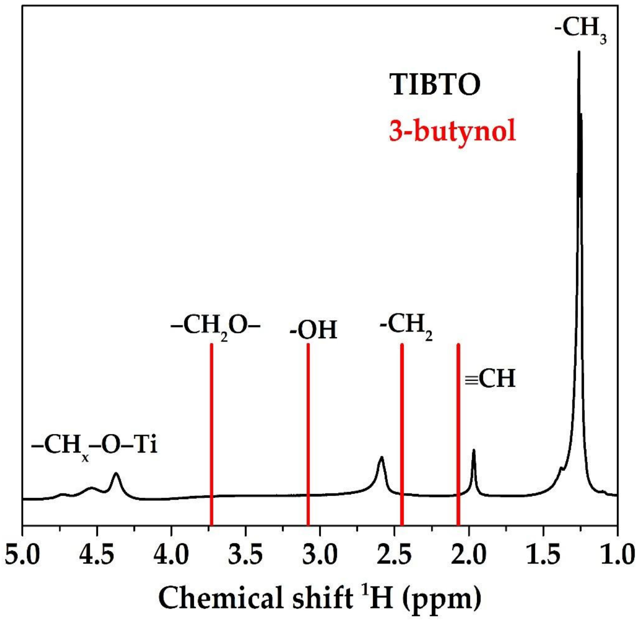

Figure S1. ^{1}H-NMR spectrum of the intermediate molecular dimer $[Ti(TIP)_3(BTO)(OH)_2]_2O$ in $CDCl_3$ solution at room temperature. Chemical shifts (ppm, relative to tetramethylsilane TMS). Red lines indicate the chemical changes of 3-butyn-1-ol.

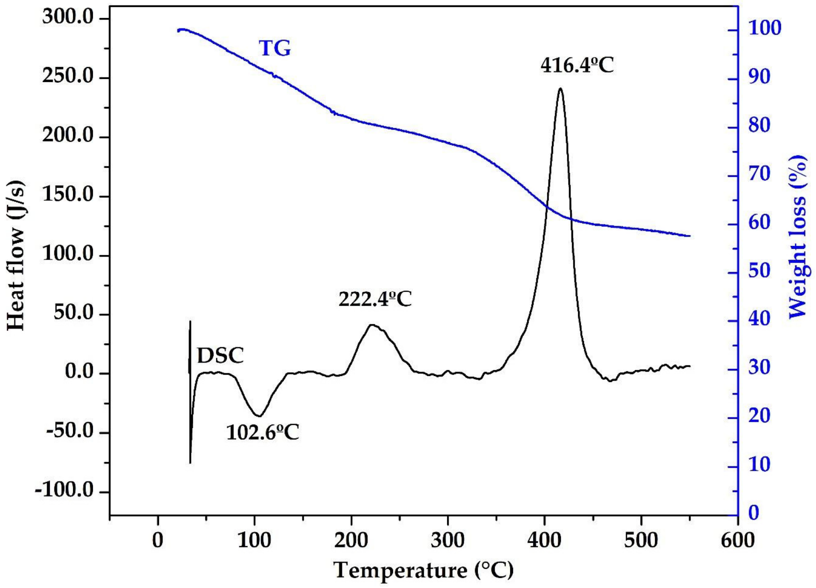

Figure S2. Thermal analysis of TiBTO. Left axis, DSC analysis; right axis, TG analysis.

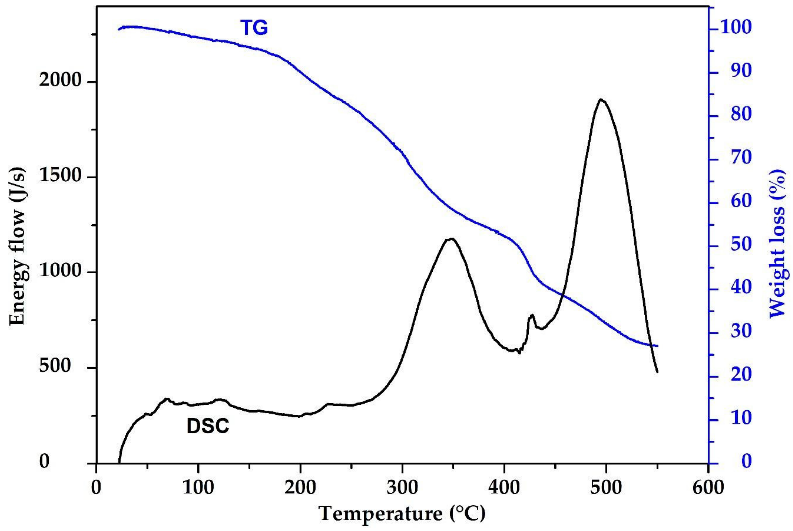

Figure S3. Thermal analysis of TiPA–A. Left axis, DSC analysis; right axis, TG analysis.

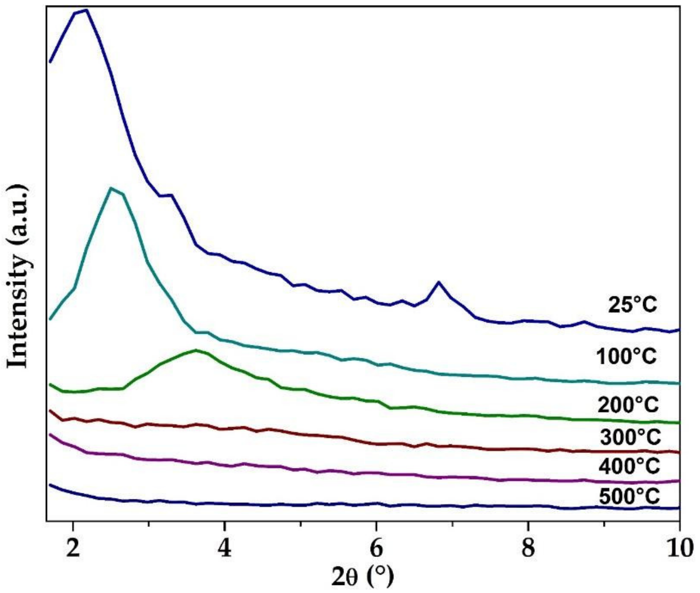

Figure S4. XRD patterns of TiPA–A samples heated in air at different temperatures.

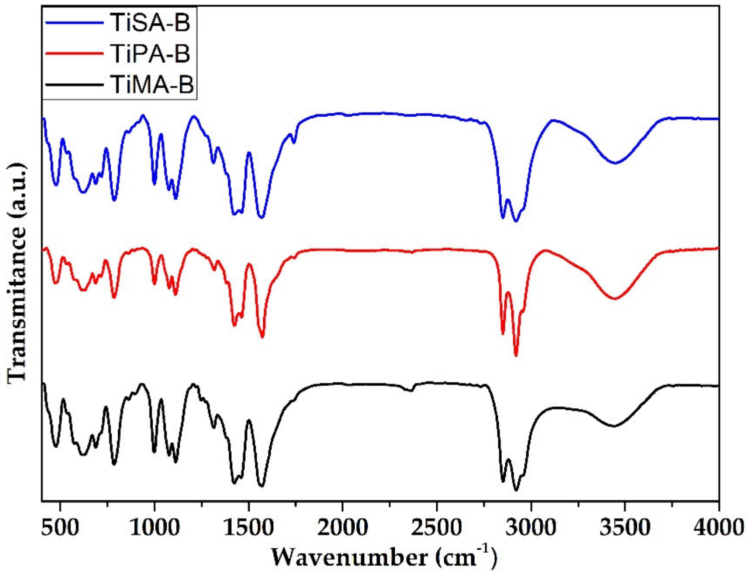

Figure S5. FTIR spectra of TiO_2@fatty-acid nanocomposites prepared through the concurrent process. TiMA–B (a); TiPA–B (b); and TiSA (c).

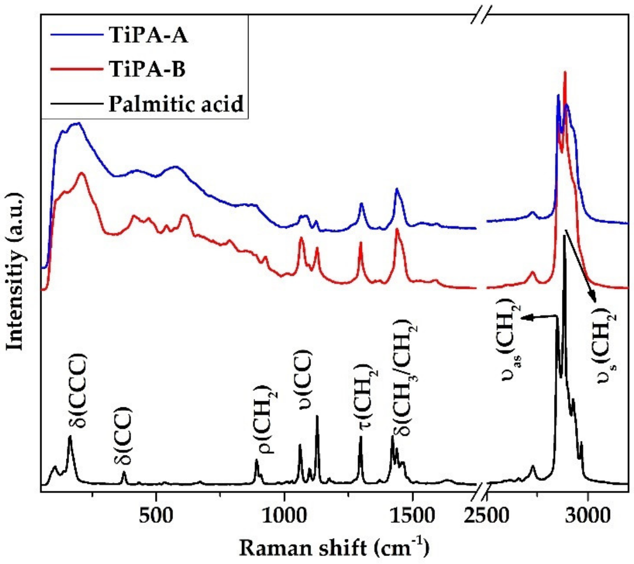

Figure S6. Raman scattering in the range of 1200 to 3200 cm^{-1} for TiMA–A, TiMA–B and pristine myristic acid.

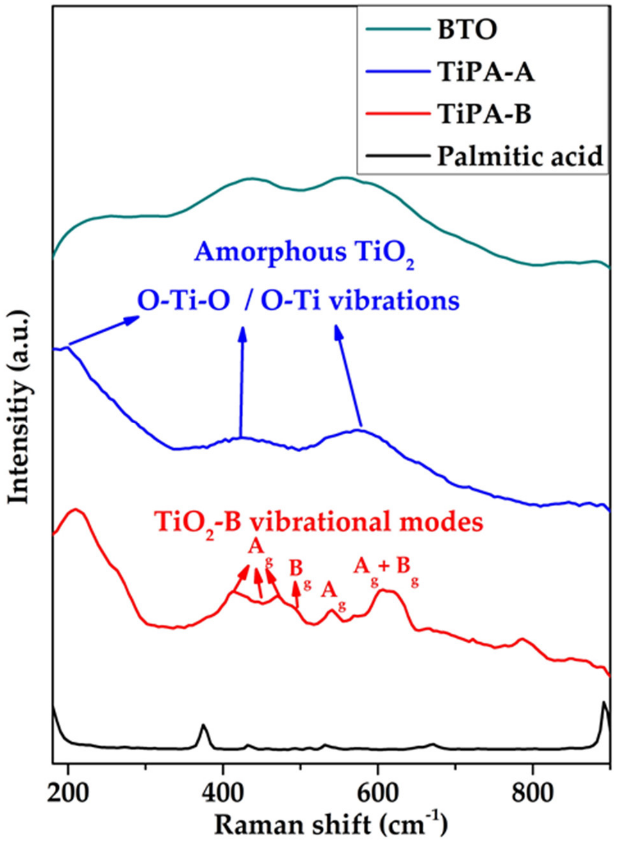

Figure S7. Raman scattering in the range of 0 to 950 cm^{-1} for TiPA–A, TiPA–B, and TiBTO and palmitic acid samples.

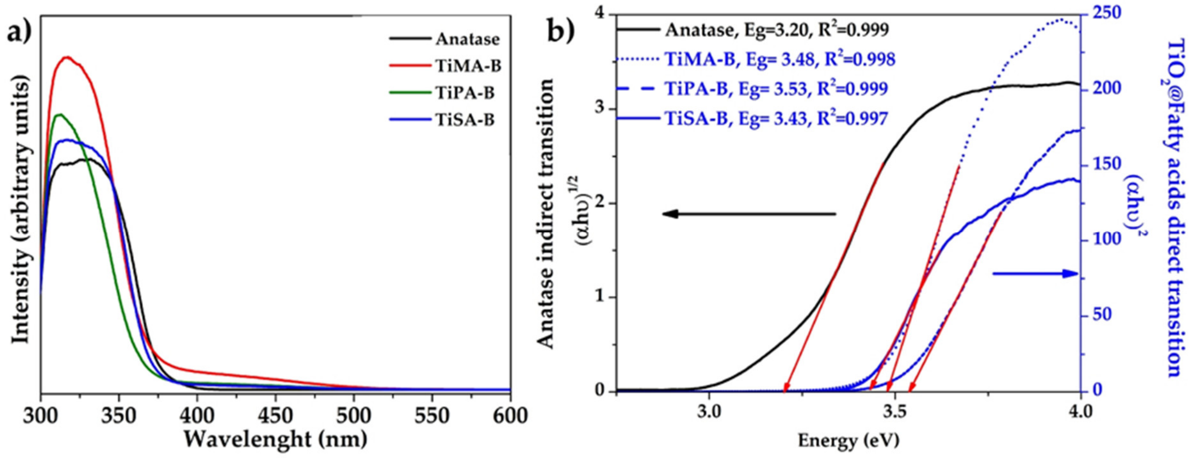

Figure S8. Absorbance spectra of TiFA-B semiconductor nanocomposites and of anatase (**a**); band gaps of TiFA–B semiconductor nanocomposites for a direct transition (right axis) compared to that of TiO_2–anatase for an indirect transition (left axis), calculated by the Tauc relationship. M (TiMA): 3.48 eV, R^2= 0.998; S (TiPA): 3.53 eV, R^2= 0.999; P(TiSA): 3.43 eV, R^2=0.997 (**b**).

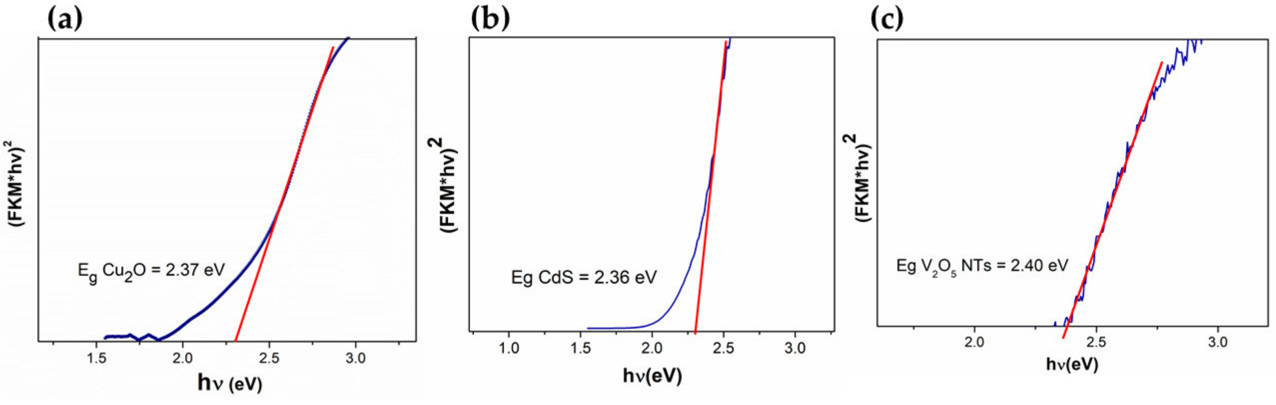

Figure S9. Tauc plots of Cu_2O (**a**), CdS (**b**), and V_2O_5 (**c**).

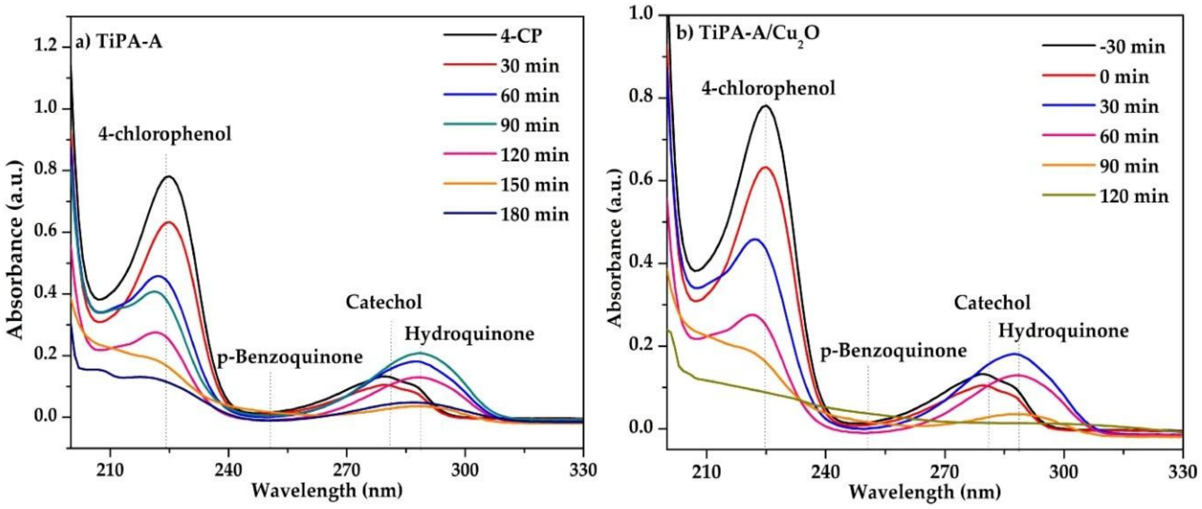

Figure S10. Simulated-solar light-driven degradation of 4–chlorophenol (4–CP) assisted by TiPA–A (**a**) and by composite TiPA–A/Cu_2O (**b**).

Table S1. Chemical composition of the products obtained by the sequential process from the analysis of microelements and complemented with thermogravimetric analysis.

Sample	Analysis Exp.(calculated) %			Empirical Formula
	C	H	Ti	
**TiBTO**	30.4 (30.50)	4.00 (3.490)	30.3 (30.44)	$TiO_2(C_4H_5)$•0.25(TiO_2 H_2O)
**TiPA-A**	49.8 (50.37)	10.2 (8.940)	17.9 (17.59)	0.7[$TiO_2(C_{16}H_{35}O)$•[$HTiO_2(C_{16}H_{36}O_2)_{2.3}$•$H_2O$]

Table S2. Chemical composition of the products obtained by the concurrent process from the analysis of microelements and complemented with thermogravimetric analysis.

Sample	Analysis Exp.(calculated) %			Empirical Formula
	C	H	Ti	
**TiMA-B**	60.81 (61.17)	10.37 (9.66)	12.67 (12.62)	$Ti(C_{14}H_{27}O)$•0.42($C_{14}H_{28}O_2$)•0.1[$HTiO_2(C_{14}H_{27}O)(H_2O)$]
**TiPA-B**	60.87 (60.91)	10,62 (11.56)	10.84 (10.85)	$Ti(C_{16}H_{35}O)$•0.4($C_{16}H_{36}O_2$)•0.4(H_2O)
**TiSA-B**	61.10 (61.00)	10.37 (11.19)	12.67 (13.09)	$Ti(C_{18}H_{39}O)$•0.03($C_{18}H_{40}O_2$)•0.3(H_2O)
